# Polish Adaptation of the Social Communication Questionnaire (SCQ) and Female Autism Phenotype: An Investigation of Potentially Sex-Biased Items in the Screening Assessment and Their Impact on Scores

**DOI:** 10.3390/brainsci11060682

**Published:** 2021-05-22

**Authors:** Agnieszka Rynkiewicz, Magdalena Szura, Daria Bernaciak, Anna Kozak, Magdalena Karwowska

**Affiliations:** 1 Department of Psychiatry, Institute of Medical Sciences, Medical College of Rzeszów University, 35-310 Rzeszów, Poland; 2 Center for Diagnosis, Therapy and Education SPECTRUM ASC-MED, 80-404 Gdańsk, Poland; 3 Students Research Club, Department of Psychiatry, Institute of Medical Sciences, Medical College, Rzeszów University, 35-310 Rzeszów, Poland; sknpsychiatriiurz@gmail.com (M.S.); dariabernaciak@gmail.com (D.B.); anna.kozak094@gmail.com (A.K.); karwowskamagd@gmail.com (M.K.)

**Keywords:** autism spectrum disorder, females, SCQ, screening, sex differences

## Abstract

Standardized screening assessments and sex differences in autism spectrum disorder (ASD) are still under-explored in Poland. This study investigated the differences between Polish ASD females and males based on the responses provided by parents/caregivers to a Polish adaptation of the Social Communication Questionnaire, SCQ Lifetime and SCQ Current. The study included 90 ASD participants from Mental Health Services and Autism Clinics in Poland with no intellectual disability and no profound communication difficulties. Parents provided information on the *SCQ* items which were compared under three domains of the Autism Diagnostic Interview-Revised (ADI-R). Four *SCQ* items with the examples were investigated. No significant differences were found between the two sexes in the three domains. The repetitive use of objects declined with age in ASD males. Although the findings of the present study did not reveal substantial gender biases in the Polish adaptation of the SCQ, it is necessary to take into account potential gender differences in the clinical presentation of ASD and in the adaptation of screening and diagnostic tools.

## 1. Introduction

Autism spectrum disorder (ASD) is no longer considered a rare neurodevelopmental condition, and its prevalence is 1 in 54 children aged eight years, according to the latest Centers for Disease Control and Prevention (CDC) estimates [1]. ASD is a lifelong condition, with symptoms usually appearing from early childhood. However, due to changes in current diagnostic criteria [2,3], an adult may also be diagnosed if they never received an ASD diagnosis in childhood [4,5,6]. Additional changes to the diagnostic criteria include the integration of the previously separate diagnostic entities of “autism” and “Asperger’s syndrome” into a single category of “autism spectrum disorder (ASD)” [2,3,7]. The presence of sensory profile abnormalities among the most recent diagnostic criteria may lead to an improved recognition rate of females with ASD [8]. Traditionally, ASD is more commonly diagnosed in males than in females across different age groups [9,10,11]. Females, however, are often misdiagnosed, diagnosed later in life, or remain undiagnosed [5,12,13,14]. A timely diagnosis, however, can reduce the difficulties and risks that females with ASD encounter over their lifetime. According to the latest estimates, autistic women are 13 times more likely to die by suicide than non-autistic women [15,16].

The sex ratio of ASD varies from 4:1 [14] to 2:1 [17], depending on the research methodology and samples, due to the population heterogeneity and comorbidities that are common in this population [18].

There are no reliable biomarkers of ASD, and assessment is based on the observation and description of core social interaction and communication characteristics, alongside the developmental history [2,3,19]. Developmental, psychological, social, and cultural factors impact autistic presentation [12,20]. The sex of an individual and the presence of psychiatric comorbidities may contribute to delaying diagnosis. For instance, women with average or above-average cognitive abilities may present with subtle symptomatology [11,21]. A unique female autism phenotype has therefore been proposed, which includes the skills of camouflaging, masking, and compensating for difficulties, but in which the set of behaviors and unusual interests traditionally linked to autism may not be observed [12]. Females may be more likely to have ASD than we currently estimate, but the traditional diagnostic criteria and assessments developed based on a male stereotype do not cover the unique pattern of behaviors and interests seen in ASD females [11,21,22].

The Social Communication Questionnaire (SCQ) [23] is a screening questionnaire completed by the parent/carer of an individual who might have ASD (henceforth, autism) [24,25]. The items are based on the algorithm of the revised version of Autism Diagnostic Interview-Revised (ADI-R) [26] and autism diagnostic criteria. The development of the SCQ, as well as other standardized assessments such as the Autism Diagnostic Observation Schedule (ADOS-2) [27] and ADI-R [26], was based on research undertaken with predominantly male samples. Furthermore, the standardization studies tended to be dominated by autistic male samples. As ASD traits vary by age and sex, this may have given rise to assessments not sensitive enough to detect the clinical presentations of autistic females [4,21,22,28,29,30]. Therefore, the present study aims to assess the potential gender bias, that is, the presence of items that exclusively concern the male autistic phenotype, in the Polish adaptation of the Social Communication Questionnaire (SCQ) [23].

## 2. Materials and Methods

Our study group consisted of 90 Polish participants (30 females and 60 males) with ICD-10-based [31] clinical diagnoses of “autism” and “Asperger’s syndrome” (the data were collected before the integration of these previously separate diagnostic categories into the singular “autism spectrum disorder” (ASD) [3] category in Poland). They all participated in our other large-scale study on ASD in females. All participants and their parents/carers were recruited from the Mental Health Services and Autism Clinics in the Pomeranian, Mazovian, and Subcarpathian regions of Poland. The inclusion criteria were: (1) age ≥ 6 years; (2) intelligence quotient (IQ) ≥ 75; (3) clinical diagnosis of “autism” or “Asperger’s syndrome” made by a psychiatrist; (4) Polish as a first and primary language; (5) no hearing or vision impairments. We included individuals with such psychiatric comorbidities as mood and anxiety disorders, attention-deficit hyperactivity disorder (ADHD), eating disorders, etc., as they are common in ASD. These additional diagnoses were verified via patient medical records, as was their performance IQ. The participants had no intellectual disabilities (ID), as confirmed via the Wechsler Intelligence Scale for Children-Revised (for verbal children and adolescents) [32] and the Wechsler Adult Intelligence Scale (for verbal adult participants) [33]. These data are presented in Table 1 along with the psychiatric comorbidities of this sample. Among the participants, there were 53 children (aged 6–11), 21 adolescents (aged 12–18), and 16 adults (aged 19–44). The participants’ parents/carers were requested to complete the Social Communication Questionnaire (SCQ) [23].

The SCQ is a 40-item parent/carer-reported screening questionnaire that concerns the symptomatology associated with autism. There are two forms, SCQ Lifetime and SCQ Current, that both contain the same items, except for items 20 to 40 of the SCQ Lifetime, which focus on the period between the child’s fourth and fifth birthdays. The SCQ Lifetime addresses the individual’s entire developmental history, while the SCQ Current concerns the individual’s behavior in the last 3-month period. The items, presented in a *YES/NO* format, are designed to be understandable to non-professionals so that they can be answered with minimal inference. The *SCQ* can be used to evaluate anyone over 4 years old whose mental age exceeds that of a 2-year-old [34].

Since the SCQ was designed based on the ADI-R algorithm, we investigated all the SCQ items within the three domains of ADI-R: (1) reciprocal social interaction, (2) communication, and (3) restricted, repetitive, and stereotyped patterns of behavior. Items 9, 10, 19, 26, 27, 28, 29, 30, 31, 32, 33, 36, 37, 39, and 40 refer to reciprocal social interaction. Items 2, 3, 4, 5, 6, 20, 21, 22, 23, 24, 25, 34, and 35 refer to communication, whereas items 7, 8, 11, 12, 13, 14, 15, and 16 refer to restricted, repetitive, and stereotyped patterns of behavior. We also specifically focused on SCQ items 11, 12, and 13, which include very specific examples (e.g., traffic lights, drainpipes, timetables, trains, dinosaurs, or spinning the wheels of a car), as well as item 14, which refers to unusual sensory interests, as presented in Table 2.

Both versions of the SCQ were completed by parents/caregivers in person or during an online (e.g., Zoom or Skype) session in the presence of a researcher. A self-stamped and addressed envelope was provided so that they could return the SCQ pen/pencil form to the researchers after the online meeting. For the purposes of this article, only the results of the SCQ administered during the screening process of a larger study have been analyzed.

The research adhered to the Declaration of Helsinki and was approved by the Research Ethics Committee of Rzeszow University (№ 8/6/2017). Informed written consent was obtained from all parents/carers and participants where appropriate, in accordance with the procedures of the above Research Ethics Committee. The Western Psychological Services (WPS) authorized the researchers to use the author-reviewed Polish research translations of the SCQ Lifetime and SCQ Current, and to reproduce the translations via paper/pencil with hand-scoring, for the sole purpose of conducting this registered academic study. Each reprint of the translated SCQ Lifetime and Current forms bore the required copyright notice that was provided by the WPS in English and Polish [34].

The statistical analysis was carried out based on the mean values of the relevant parameters in order to determine differences that could be directly attributed to sex and age. Statistical comparisons were made using a series of non-parametric Mann–Whitney U tests. All statistical analyses were carried out using SPSS software (IBM SPSS Statistics v. 25).

## 3. Results

We analyzed the results from four SCQ items (11, 12, 13, and 14) in order to determine significant differences in Lifetime and Current responses between females and males. The results are presented in Table 2. Table 3 shows the results for the same items in three age groups. The statistical analysis included a series of non-parametric tests due to the small-sized samples, which did not guarantee exact normality. As such, we used McNemar’s test for a within-group pre–post comparison of the dichotomous dependent variables.

There was only one significant difference between the Current and Lifetime results in males, and this was for item 12. The positive response rate to that item in the SCQ Lifetime was 22.0% (*n* = 13), as compared to 35.6% (*n* = 21) in the SCQ Current (*p* = 0.039). There were no significant changes between the Current and Lifetime answers in all age groups.

**Figure 1:**
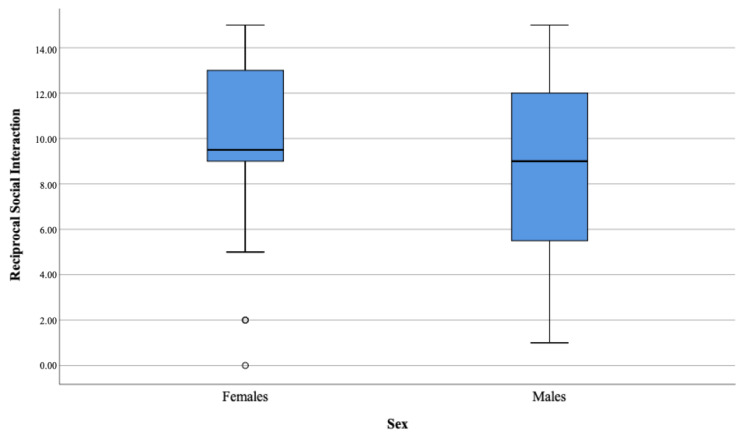
Reciprocal Social Interaction domain.

**Figure 2:**
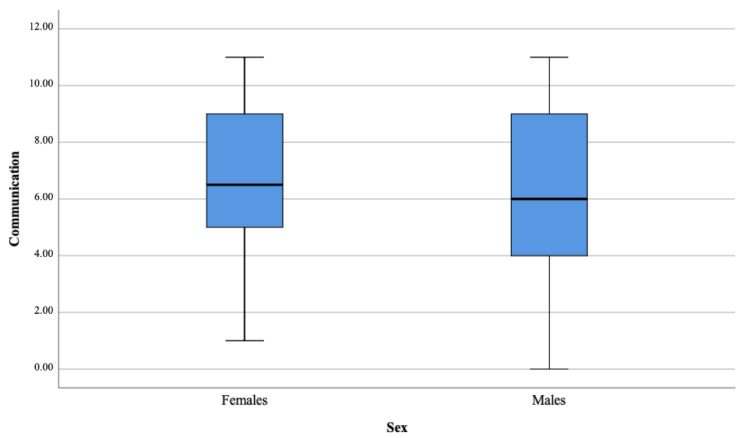
Communication domain.

**Figure 3:**
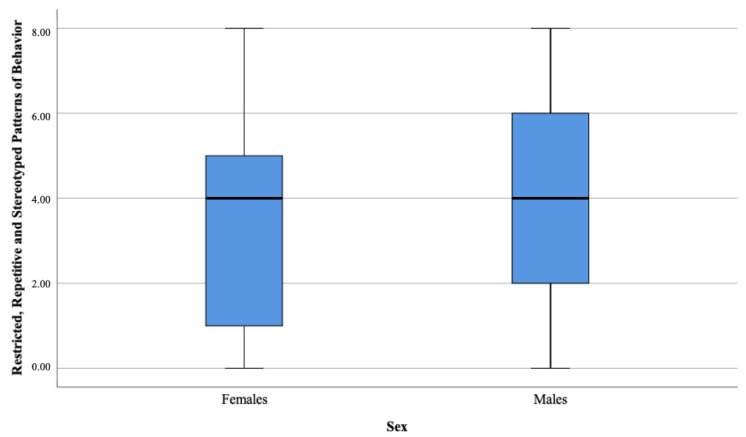
Restricted, repetitive, and, stereotyped patterns of behavior domain.

**Figure 4:**
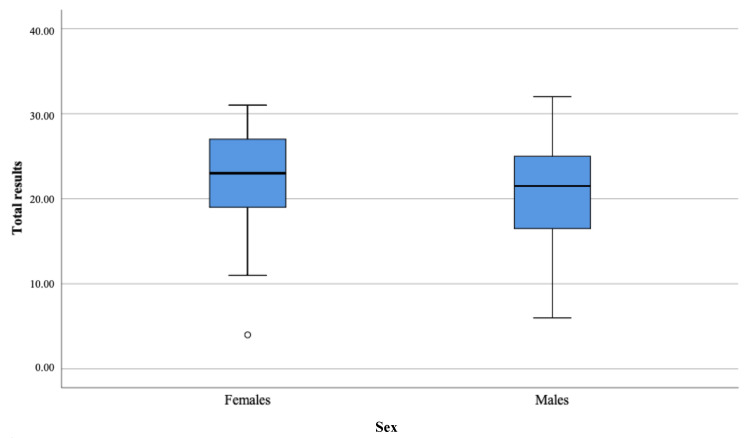
Total results from the three domains.

There were no significant differences in the three analyzed domains between ASD females and ASD males as presented in Table 4 and Figure 1, Figure 2, Figure 3 and Figure 4.

Table 5 presents examples of special interests provided by parents/carers of ASD females in response to SCQ items 11 and 13, categorized by the researchers.

## 4. Discussion

In our study, no significant differences between ASD males and females were found after grouping the SCQ items into the three domains of the ADI-R. The study showed that the ASD females in our group were phenotypically similar to ASD males. The results cohere with those of a recent large-scale and multi-site analysis by Kaat and colleagues [35], wherein no differences were found between autistic males and females assessed via the ADI-R, and consequently via the SCQ, the latter of which is based on the former. The results are also in line with those of Park and colleagues [36], who found that normative sex differences may be absent in children with ASD. Much like the patients in Kaat’s [35] or Park’s [36] analyses, all the individuals in our study had clinical ASD diagnoses as well. Furthermore, we hypothesize that narrow constructs and a wide range of behavioral exemplars might elucidate more subtle sex differences among the individuals already diagnosed with ASD. Such an approach has already been proposed by Lai and colleagues [37] and Wood-Downie and colleagues [38]. Thus, we investigated the SCQ items where the behavior exemplars are included and might have guided the parents in their responses. Although there were no significant differences in terms of three SCQ items (11, 13, and 14) between ASD males and females in our sample, there was a difference in item 12, which concerns the repetitive use of objects. This trait was reported to decline with age in ASD males. Parents of ASD males reported their child’s repetitive use of objects in the past, but not in the present, thus indicating a change. The results of this single SCQ item should be interpreted cautiously. It should be analyzed further in the future, not only against the detailed data obtained from, for example, a structured developmental interview such as the ADI-R, but the interpretation should also incorporate previous research undertaken with the ADI-R, given that the SCQ questions are based on the ADI-R. SCQ item 12 belongs to the restricted and repetitive behavior (RRB) domain under the ADI-R. The observed reduction in repetitive behaviors coheres with the previous research on RRB in general ASD [39]. However, the current result concerns only one single SCQ item out of the eight items that are listed under the RRB domain of ADI-R. It has also only been reported in ASD males. This finding may be dependent on the sex and age of the participants in our study. Parents may not report their adult child’s repetitive use of objects because the ADI-R (and consequently the SCQ) may not capture essential characteristics of ASD adults, as has already been reported by Lai and Baron-Cohen [24] and Fusar-Poli and colleagues [40], especially for females with high cognitive abilities. There is a possibility that ASD females with high cognitive abilities (such as the subjects in our study) develop camouflaging or compensating strategies over time, from childhood to adulthood, so as to mask the core symptoms of ASD. There may also be a recall bias in the parents of ASD adults, as there are many years between childhood (half of the questions of the SCQ Lifetime cover the ages of 4 to 5 years old) and the current time of assessment in adulthood [23].

That being said, this study yielded additional data regarding the four abovementioned SCQ items that we consider important. All parents/carers of ASD females who observed preoccupying and intense special interests in their children, and as a result responded YES to the SCQ items 11, 12, and 13, provided additional comments. The parents of ASD females commented that the special interests of their children were different from the examples provided in the SCQ. They commented either verbally or wrote their own examples next to the relevant SCQ items. The examples provided of female ASD children’s special interests (presented in Table 5) are not traditionally linked to autism. The comments included interests in different animals, fairytales, arts, languages, human or animal behavior, literature, theatre, dance, as well as beauty and fashion. All comments were annotated for the purpose of this study. Including such interests in the screening assessment was important to the parents/carers of ASD females, as they highlighted the lack of relatable examples in the form. It is advisable to include such behavioral exemplars in the screening assessments so as to more accurately capture female autistic traits and to ensure the examples provided in the SCQ also relate to females. We decided to report these additional clinical data here so as to raise additional scientific questions.

In our study, 63 (70.0%) subjects were found to have at least one psychiatric comorbidity. The prevalence of psychiatric comorbidities in this study group is presented in Table 1. Mood disorders, such as depression or BD, were more common in ASD females. There were no cases of eating disorders in ASD males, as compared to two cases in ASD females. The clinical data raise additional questions as to the impact of comorbidities on SCQ scores. For example, anxiety disorders, depression, or ADHD can present as functional and behavioral difficulties and can affect the social interaction domain, while OCD, tic disorders, or eating disorders can be reflected in the RRB domain. Additionally, the presence of medication use and other comorbid medical conditions, such as epilepsy, gastrointestinal (GI) disorders, allergies, etc., all of which are common in ASD, could also influence the SCQ scores. However, the influences of comorbid diagnoses and medical treatments on the SCQ scores of the Polish sample might require further research. This offers an interesting suggestion for future investigations.

All parents/carers in our study who responded to the SCQ via online meetings (e.g., Zoom or Skype) said this modality was convenient. The use of the SCQ as an online pre-assessment could benefit many families awaiting an ASD evaluation for their child. Unfortunately, the SCQ is only available for research purposes in Poland, but it has been extensively used for years in other European countries in both research and clinical practice. The SCQ online evaluation system is available in the USA, presented by the WPS [41], which is an advantage during the COVID-19 pandemic when personal contact carries the risk of infection. 

It is important to acknowledge some limitations while discussing the results of the present paper. The sample size was small, especially for age-based clustering into children, adolescents, and adults. We were also limited by the inclusion criteria of the larger project. The ASD population is very heterogenous, with possible comorbid intellectual disability (ID), but there were no such individuals in our sample. Some parents struggled with the YES/NO format of the SCQ in this study. Similar limitations have been reported before, for example by Frazier and colleagues [42] and Evans and colleagues [43].

The study also highlights the benefits of the online SCQ pre-assessment, especially in such countries as Poland where the SCQ is currently implemented. It helps many families awaiting an ASD diagnostic assessment for their child. Examples of best practice with the SCQ are particulalry relevant during the COVID-19 pandemic, as face-to-face appointments have been limited or sometimes not possible at all.

## 5. Conclusions

No significant differences were found between the two sexes in the three domains. The repetitive use of objects declined with age in ASD males. Autistic females presented with unusual and intense interests, covering a variety of topics and themes that are not traditionally linked to autism. Such interests are not included as examples in the SCQ. This study confirms that comorbidities in ASD individuals are common. It is advisable to replicate this research in the future with larger samples, including autistic individuals with intellectual disabilities (ID), and comparing them with control groups of non-autistic individuals with matching comorbidities. Although the findings of the present study did not reveal substantial gender biases in the Polish adaptation of the SCQ, it is necessary to take into account potential gender differences in the clinical presentation of ASD and in the adaptation of screening and diagnostic tools. This study contributes to a further understanding of autism spectrum disorder in females.

## Figures and Tables

**Table 1:** Demographics including the IQ scores, age, and psychiatric comorbidities.

	Participants with ASD (*N* = 90)		
Sex		Females (*N* = 30)	Males (*N* = 60)
IQ	Mean (SD)	108 (12.27)	102.62 (12.53)
Age	Mean (SD)	15.93 (11.09)	11.4 (5.85)
Comorbidity	With comorbidity	19 (63.3%)	44 (73.3%)
	Without comorbidity	11 (36.7%)	16 (26.7%)
Type of comorbidity	Mood (affective) disorders: depression, BD	3 (15.8%)	4 (9.1%)
	Neurotic, stress-related, and somatoform disorders: anxiety, OCD, phobias, agarophobia, panic disorder	10 (52.6%)	13 (29.5%)
	Disorders with onset occuring in childchood or adolescence: ADHD, ODD, tic disorder	2 (10.5%)	17 (38.6%)
	Psychological development: dyslexia, dyspraxia	1 (5.3%)	7 (15.9%)
	Syndromes associated with psychological disturbance: ED	2 (10.5%)	0 (0.0%)
	Others: adult personality or substance-related disorders	1 (5.3%)	3 (6.8%)

*N*—number of participants included in the sample; SD—standard deviation. Diagnostic categories are grouped and based on the ICD-10, as retrieved from patient medical records. BD—bipolar affective disorder; OCD—obsessive–compulsive disorder; ADHD—attention-deficit hyperactivity disorder; ODD—oppositional defiant disorder; ED—eating disorder.

**Table 2:** McNemar’s tests results: comparison of the SCQ Lifetime to Current by sex.

Item	Sex	Current (*n*)	Lifetime (*n*)		*p*
			No	Yes	
11. Has she/he ever had any interests that preoccupy her/him and might seem odd to other people(e.g., **traffic lights, drain pipes, or time tables**)?	Female	No	17	1	1.000
		Yes	0	12	
	Male	No	23	2	1.000
		Yes	2	33	
12. Has she/he ever seemed to be more interested in parts of a toy or an object (e.g., **spinning the wheels ** **of a car**), rather than using an object as it was intended?	Female	No	22	1	1.000
		Yes	2	5	
	Male	No	36	2	0.039
		Yes	10	11	
13. Has she/he ever had any special interests that were unusual in their intensity but otherwise appropriate for her/his age and peer group (e.g., **trains, dinosaurs**)?	Female	No	17	0	1.000
		Yes	1	12	
	Male	No	19	4	0.754
		Yes	6	31	
14. Has she/he ever seemed to be unusually interested in the sight, feel, sound, taste, or smell of things or people?	Female	No	7	1	0.625
		Yes	3	19	
	Male	No	11	2	1.000
		Yes	14	44	

**Table 3:** McNemar’s tests results: comparison of the SCQ Lifetime to Current by the age groups.

Item	Age	Current (*n*)	Lifetime (*n*)		*p*
			No	Yes	
11. Has she/he ever had any interests that preoccupy her/him and might seem odd to other people (e.g., **traffic lights, drain pipes, or time tables**)?	Child	No	27	1	1.000
		Yes	2	26	
	Adolescent	No	8	1	1.000
		Yes	0	10	
	Adult	No	5	1	1.000
		Yes	0	9	
12. Has she/he ever seemed to be more interested in parts of a toy or an object (e.g., **spinning the wheels of ** **a car**), rather than using an object as it was intended?	Child	No	37	2	0.289
		Yes	6	11	
	Adolescent	No	14	1	1.000
		Yes	2	2	
	Adult	No	7	0	0.125
		Yes	4	3	
13. Has she/he ever had any special interests that were unusual in their intensity but otherwise appropriate for her/his age and peer group (e.g., **trains, dinosaurs**) ?	Child	No	26	3	0.727
		Yes	5	22	
	Adolescent	No	5	1	1.000
		Yes	2	11	
	Adult	No	5	0	1.000
		Yes	0	10	
14. Has she/he ever seemed to be unusually interested in the sight, feel, sound, taste, or smell of things or people?	Child	No	7	1	0.375
		Yes	4	42	
	Adolescent	No	6	2	1.000
		Yes	1	10	
	Adult	No	5	0	1.000
		Yes	1	9	

**Table 4:** Differentiation between ASD females and ASD males in terms of the studied variables–three domains.

	Females (*n* = 30)				Males (*n* = 60)				Z	*p*	η2
	Mean Rank	M	Me	SD	Mean Rank	M	Me	SD			
Reciprocal Social Interaction	49.88	9.67	9.50	3.80	43.31	8.55	9.00	4.24	−1.13	0.258	0.01
Communication	49.73	6.73	6.50	2.75	43.38	6.07	6.00	2.85	−1.10	0.273	0.01
Restricted, Repetitive, and Stereotyped Patterns of Behavior	40.62	3.57	4.00	2.54	47.94	4.28	4.00	2.31	−1.26	0.206	0.02
Total	50.20	21.97	23.00	6.10	43.15	20.63	21.50	5.71	−1.21	0.227	0.02

**Table 5:** Parents/carers’ comments and annotations on the special interests of their ASD female child.

Age	Parents/Carers’ Comments and Annotations	Category
7 y.o.	She is interested only in cat breeds identification	Animals
9 y.o.	She collects makeup products	Fashion and beauty
17 y.o.	She is obssessed about horses and spends long hours researching all about horses on the internet	Animals
8 y.o.	She is an avid reader, and she always takes her book with her when she leaves the house	Literature
15 y.o.	She insists on going to the same play several times	Theatre and dance
6 y.o.	She insists on reading books only about fairies, collects only fairy dolls, and goes out only dressed as a fairy	Fairytales
19 y.o.	All she wants to learn is Italian, she repeats new phrases all the time, and she already speaks two other foreign languages fluently	Languages
13 y.o.	She knows everything about Gustav Klimt’s art	Arts
32 y.o	She is interested only in autism in females, writes a blog about it, attends meetings as a self-advocate, talks only about this topic if you let her	Human behaviour
9 y.o	She only talks about dog’s behaviour, how to train dogs, knows the addresses of every dog training center and pet shop in the area, and insists on going there in any spare moment	Animal behaviour

## Data Availability

The data supporting reported results are available upon reasonable request from the corresponding author. They are held by the co-author’s institution: Department of Psychiatry, Medical College of Rzeszow University in Poland.

## Author’ Information

A.R. is an internationally recognized, certified trainer in the reliable administration of ADOS-2 and the INSAR Global Senior Leader for Poland (2021–2022).
